# A Rare Variation of the Levator Scapulae Muscle: A Case Report and Review of Literature

**DOI:** 10.7759/cureus.42355

**Published:** 2023-07-24

**Authors:** Lyubomir L Gaydarski, Georgi P Georgiev, Łukasz Olewnik, Piotr Karauda, Boycho Landzhov

**Affiliations:** 1 Department of Anatomy, Histology and Embryology, Medical University of Sofia, Sofia, BGR; 2 Orthopaedics and Traumatology, University Hospital Queen Giovanna - ISUL, Sofia, BGR; 3 Department of Anatomical Dissection and Donation, Medical University of Lodz, Lodz, POL

**Keywords:** neck surgery, clinical significance, neck's posterior region, anatomical variations, levator scapulae muscle

## Abstract

Muscle variations in the posterior neck region are mainly categorized as variations in the origin and insertion of the muscles and the presence of accessory slips or rudimentary muscles. The levator scapulae muscle is a variable muscle with several different types of variations described throughout the literature. Herein, we report a rare unilateral case of an accessory slip from the levator scapulae. Aberrant muscle slip originates from the distal one-third of the levator scapulae. Then, it passes upwards and outwards above a vascular bundle containing a superficial branch of the transverse cervical artery and vein. The deviant muscle slip inserts onto the superior nuchal line. Muscle variations in the neck's posterior region and the levator scapulae's variations, as per se, have the utmost clinical significance since they might be mistaken for tumor mass. Moreover, such variations might be deceptive during surgical procedures in the region. Therefore, detailed knowledge of such complex muscular variations in the posterior region of the neck is paramount for surgeons and radiologists alike.

## Introduction

The muscles of the posterior region of the neck have complex topographical relations, and based on their origin and insertion points, they might be involved in the movement of several joints. From a functional standpoint, this group of muscles is mainly responsible for the posture maintenance of the head and movement of the cervical and thoracic regions. Moreover, levator scapulae (LS) and the rhomboid muscles (RM) take part in shoulder movement [1]. The LS typically originates from the transverse processes of C1-C4 with an average of four slips that descend posterolaterally and insert on the superomedial border of the scapula [1]. Muscle deviations in the region are seldom found incidentally during routine anatomical dissections. Variations in the LS are mainly portraited by variability in their origin points or accessory muscle slips inserted into adjacent muscles [2,3].

Herein, we present a rare case of an aberrant branch arising from the LS, ascending outward and passing above a vascular bundle comprised of superficial branches of the transverse cervical artery and vein. The deviant muscle slip then inserts onto the superior nuchal line (SNL).

## Case presentation

**Figure 1:**
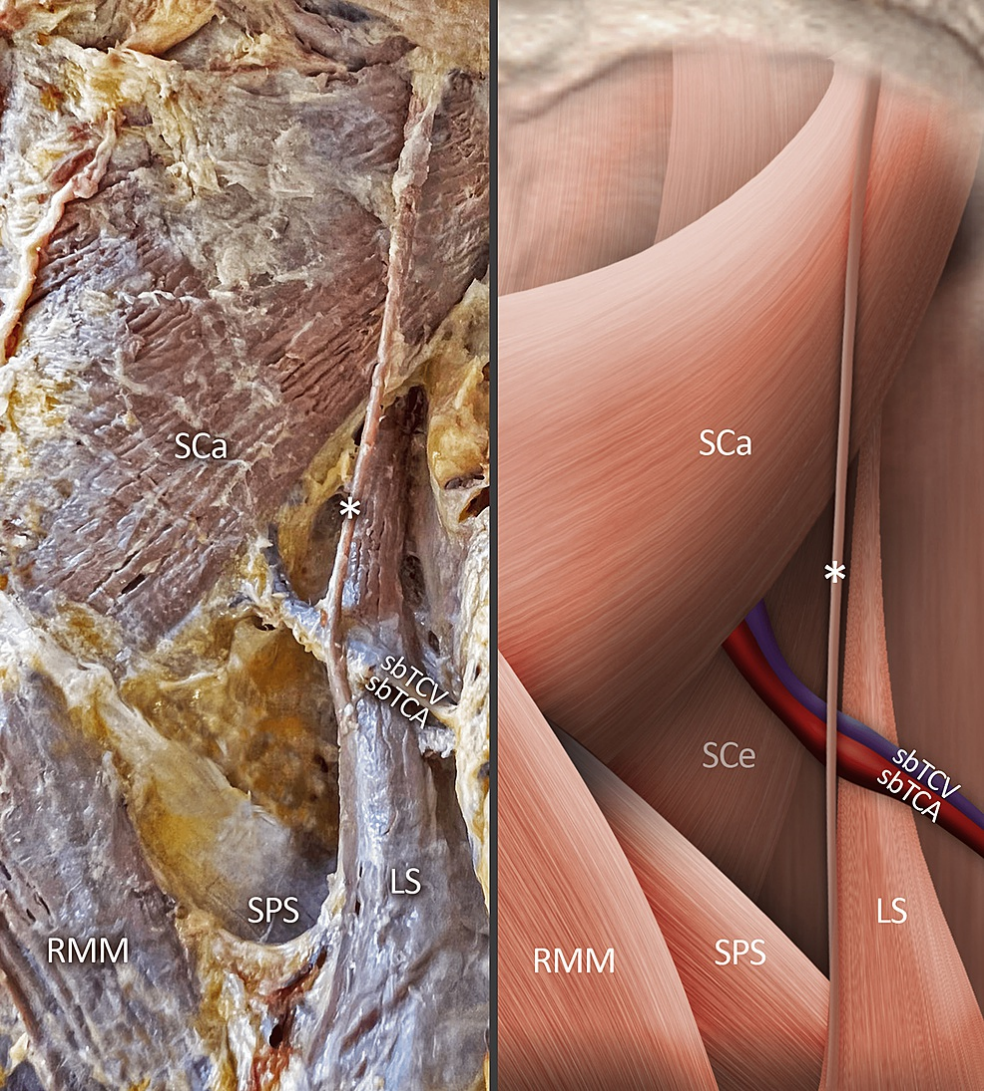
Photograph and schematic view of the presented variant muscle slip The presented variant muscle slip that started from the levator scapulae, crossing over the superficial branches of the transverse cervical artery and vein and then inserted into the superior nuchal line. SCa - splenius capitis muscle; SCе - splenius cervicis muscle; RMM - rhomboid minor muscle; LS - levator scapulae muscle; SPS - serratus posterior superior muscle; variant muscle - asterisk; superficial branch of the transverse cervical artery - sbTCA and vein - sbTCV.

A scarce and intriguing variation was noted during a standard dissection of a 76-year-old formalin-fixed cadaver with an educational purpose for the practical seminars of medical students at the Anatomy, Histology, and Embryology Department, Medical University of Sofia. With the progression of the dissection, a slander muscle slip was discovered on the right posterior neck region beneath the nuchal fascia. This aberrant muscle slip originated from the distal one-third of the muscle body of the LS, passing upward, initially inward, surrounding a vascular bundle comprised of superficial branches of the transverse cervical artery and vein. Then, the muscle slip continuously to ascend outward, laterally, and above the splenius capitis before inserting into the SNL between the splenius capitis and the sternocleidomastoid (Figure 1).

Мeasurement of the aberrant muscle slip was taken with a standard ruler. The muscle slip is 86 mm long and 5.3 mm thick. The blood supply to the aberrant muscle slip was carried via a branch of the ascending branch of the transverse cervical artery, and the innervation was by a branch of the dorsal scapular nerve. No such aberrant muscle slip was found on the left LS. No medical or surgical history of the cadaver was available, and no scars were observed prior to the dissection.

## Discussion

The LS is an important topographical landmark in the posterior region of the neck. Knowledge of such muscular deviation is intriguing from an anatomical standpoint and has high clinical relevance since it might pose a misleading landmark during surgical procedures. According to the literature, the variations in LS can be divided into two categories. The first type of variation concerned the origin of LS from different cervical vertebrae: C1-C2 (3.3%); C1-C3 (26.6%); C1-C4 (66.6%); and C1-C5 (3.3%) [4]. The second type comprises anomalous (accessory) slips: usually, the LS splits into two parts and seldom into three. Aberrant slips were recorded to insert onto the splenius capitis, longissimus cervicis, serratus posterior superior, rhomboideus major and minor, scalene, and trapezius muscles [5]. Furthermore, Humphry GM reported a slip that passed across the subclavian artery and was inserted into the subclavius muscle [5]. Macalister A reported three muscle slips arising from the transverse processes of C3-C5 and fusing before attaching to the scapula [6]. Furthermore, Macalister A reported that in rare instances, accessory slips might extend to the temporal bone, occipital bone, or mastoid process [6]. Wood described slips from the LS fusing with the scalenus medius, splenius capitis, and splenius cervicis [7]. Macbeth and Martin reported a case of aberrant muscle slip originating from the mastoid process and fusing with the C1 band of the LS [8]. Loukas et al. described an accessory head of the LS inserted onto the nuchal ligament, rhomboid major, and the serratus posterior superior [9]. In a recent study, Chotai et al. reported an aberrant muscle slip originating from the bulk of the LS ascending outward and inserted into the mastoid process [10]. Au et al. performed an MRI-based study and found a 43% incidence rate of variations in LS [3].

Another rare variation in the posterior region of the neck is the occipitoscapularis muscle (known as rhombideus capitis). This deviant muscle has only been reported a few times throughout the literature. Wood first described the muscle as arising from the occipital bone next to the splenius capitis muscle, below the origin point of the trapezius on the SNL [7]. Knott (1883) and Rogawski (1990) reported cases of a muscle with cranial attachments to the medial one-third of the SNL right above the occipital attachment of the semispinalis capitis muscle [11,12]. They named this muscle the rhomboideus occipitalis. The variant muscle descended laterally, passing between the splenius and trapezius before attaching to the medial border of the scapula via a short tendon. Rhomboideus capitis was reported to have muscular slips extending to the splenius, levator scapulae, serratus posterior superior, and serratus anterior muscles [7]. Recently, Stanchev et al. reported a case of bilaterally present occipitoscapularis muscle [13].

Nevertheless, in our case, the aberrant muscle inserts onto the SNL with no relation to the insertion of the splenius capitis. It descends outward and fuses with the distal third of the levator scapulae, thus comprising a scarce variant of the LS. The exact incidence rate of such rare variation is yet unknown, and only a few other cases of such aberrant muscle slips from the LS arising to the SNL have been previously documented by Macalister and Meckel [2,6].

The reason for all these numerous muscle variations is yet unknown. However, the key to understanding why such variations are present lies in embryological development. The body musculature originates from the somites, whereas the head muscles originate from the cranial mesoderm. The presence of lateral occipital mesoderm as a source for vertebral neck musculature and the transitional location of neck muscles further highlight the sophisticated embryological development of the neck muscles. Thus, muscle variations in the posterior neck region might result from disturbance during the embryological period [14].

Additional slips of the LS may further add to the potential for developing neck or shoulder pain. Moreover, levator scapulae syndrome manifests with radiating pain in the scapular region, emitted toward the neck and shoulder. The exact etiology of this syndrome is yet unknown. However, muscle variations are a plausible cause [15].

Furthermore, according to Erro et al., recent studies link LS with the pathogenesis of torticollis [16]. LS might be the sole reason for torticollis, and the presence of additional muscle slips might lead to the deterioration of the condition [16]. Therefore, knowledge of such variations in the LS is essential for identifying the reason behind the torticollis and the key to its correct treatment. Moreover, the LS and the tendons of the RM are essential for the Eden-Lauren procedure, used for the surgical treatment of trapezoid palsy. This procedure transfers the LS tendon to the acromion, whereas the RM is attached to the infraspinatus fossa [17].

From a radiological standpoint, detailed knowledge of such supernumerary muscle variations is essential since these muscles might easily be mistaken for tumors [18]. In addition, there are cases of palpable neck masses treated surgically, which turned out to be hypertrophy of the ipsilateral LS. Furthermore, Shpizner and Holliday reported that sometimes LS with normal morphology might be mistaken for a tumor mass due to muscular atrophy on the contralateral LS [19].

## Conclusions

The present article reports a rare LS variation and highlights its clinical significance. It might pose a misleading landmark during neck surgery or get misinterpreted as tumour mass during a radiological examination. Furthermore, such muscular variation might lead to the manifestation of the levator scapulae syndrome.
